# Alginate–Chitosan Membranes for the Encapsulation of Lavender Essential Oil and Development of Biomedical Applications Related to Wound Healing

**DOI:** 10.3390/molecules28093689

**Published:** 2023-04-25

**Authors:** Encarnación Cruz Sánchez, María Teresa García, Joana Pereira, Filipe Oliveira, Rita Craveiro, Alexandre Paiva, Ignacio Gracia, Jesús Manuel García-Vargas, Ana Rita C. Duarte

**Affiliations:** 1 Department of Chemical Engineering, Facultad de Ciencias y Tecnologías Químicas, University of Castilla-La Mancha, Avda. Camilo José Cela 12, 13071 Ciudad Real, Spain; 2 LAQV-REQUIMTE, Chemistry Department, NOVA School of Science and Technology, 2829-516 Caparica, Portugal

**Keywords:** wound healing, membranes, alginate, chitosan, lavender essential oil, water uptake ability, cell viability, biocompatibility assessment

## Abstract

Biopolymers such as chitosan (CHT) or alginate (ALG) are among the most prominent for health-related applications due to their broad bioactivity. Their combination for the preparation of membranes is hereby proposed as an application for wound healing with the incorporation of lavender essential oil (LEO), widely known for its antioxidant and antimicrobial properties. The preparation of CHT, CHT + LEO, ALG, ALG + LEO, and CHT/ALG + LEO membranes was accomplished, and its composition was analyzed using Fourier Transform Infrared Spectroscopy (FTIR). The water absorption capacity and oil release profile of the membranes revealed higher water uptake capacity when a lower LEO release was obtained. The combined CHT/ALG + LEO film showed a water uptake percentage of 638% after 48 h and a maximum LEO release concentration of 42 mg/L. Cytotoxicity and biocompatibility of the prepared membranes were studied using a HaCaT cell line, with an assessment of cell viability regarding film leachables, DNA quantification, and DAPI-phalloidin staining. The results revealed that the indirect contact of the prepared membranes via its leachables does not compromise cell viability, and upon direct contact, cells do not adhere or proliferate on the surface of the membranes. Moreover, the CHT/ALG + LEO membrane increases cell proliferation, making it suitable for applications in wound healing.

## 1. Introduction

Skin is the largest organ of the human body, and its main function is to protect against external agents such as bacteria, chemicals, and different temperatures. For this reason, skin is highly susceptible to external damaging agents. Despite having remarkable regenerative properties, the skin healing process is sometimes hindered and this can even lead to chronic wounds, due to diabetes or pressure ulcers, for example [1,2]. Although this is an ongoing problem, there are very few treatments that are able to enhance the wound healing process. Currently, the treatment directions for wound care are initiation with antibiotics therapy, removal of damaged tissue or foreign objects from a wound, and application of dressings that maintain a moist environment, such as a compress [3]. Therefore, there is a need to find alternative solutions to hasten wound healing that are combined with antimicrobial and anti-inflammatory properties.

Growing environmental concerns and the problems associated with the high dependence on fossil fuels have promoted the use of natural substances, such as biopolymers, in all sectors and especially in biomedical or drug-related fields. Biopolymers are being widely used because of their lower impact in terms of pollution, which is because they are obtained from natural resources or by-products. These materials are biodegradable, biocompatible, environmentally friendly, and widely available; they are associated with low production costs; and they have inherent antimicrobial potential [4].

Among the biopolymers, polysaccharides are the most prominent in health-related applications due to their broad biological activity. They are present in different configurations: fibers, membranes, hydrogels, capsules, nanostructures, micelles, etc. The biopolymers that have been more prevalent in recent years include chitosan (CHT) and alginate (ALG), which have been widely applied for wound healing and other skin issues [5,6,7].

CHT, a natural cationic polysaccharide consisting of (1→4)-2-amino-2-deoxy-β-d-glucan, is the partially to fully deacetylated form of chitin. The functional properties of CHT, such as solubility and swelling index, are strongly influenced by the degree of acetylation, which is given by the distribution of amino groups along the polymer chain and, in this case, corresponds to a polycationic nature in acidic media. Moreover, by modifying its molecular weight, the functional properties can be controlled depending on the intended application: its solubility in water can be increased or its viscosity can be reduced [8,9]. Furthermore, CHT continues to play an important role in biomedical research due to its biological properties, such as biocompatibility or biodegradability [10], as well as its antimicrobial capacity [11], antioxidant potential [12], low toxicity, and ability to accelerate dermal regeneration [13]. Because of these characteristics, this polymer is one of the most widely used for the synthesis of hydrogels and membranes and in other applications for controlled drug release or tissue engineering [14].

ALG is a natural polysaccharide extracted from brown algae. Extraction is performed with a dilute alkaline solution, which solubilizes the alginic acid present. Free alginic acid is obtained by treating the resulting thick and viscous mass with mineral acids. The alginic acid can then be converted into a salt, sodium alginate currently being the most widely used form. Alginic acid is a linear polymer consisting of d-mannuronic acid and l-guluronic acid residues arranged in blocks in the polymer chain [15,16]. ALG has excellent bioactivity and biocompatibility characteristics, and for this reason, it is widely used for cell encapsulation and drug delivery systems [17,18]. In recent years, it has become the biodegradable material of choice in the pharmaceutical, biomedical, and, particularly, food packaging industries, due to its great ability to form membranes [19,20]. This ability, together with alginate’s antimicrobial potential [21], also makes it suitable for applications related to wound healing, in the form of hydrogels, nanofibers, or films [15].

The combination of biomacromolecules such as CHT with sodium ALG for the development of films also shows interesting advantages since the individual characteristics of each can be improved. In addition, the combination of CHT and ALG has been shown to work as membranes applied to cell regeneration and wound healing [17,22,23]. Furthermore, some studies have shown that the incorporation of some types of essential oils can improve the properties of hydrogels and membranes, mainly by increasing their antimicrobial potential, and therefore make them interesting for the treatment of skin pathologies or for food packaging [24,25,26,27,28].

Essential oils are known for their versatility and health-promoting attributes [29]. One of the most frequently used is lavender essential oil (LEO), for applications related to cosmetics, and in recent years, it has been also considered a good option to produce pharmaceuticals. This is due to the bioactivity of some of the predominant chemical compounds in its composition, such as linalool, which has a high antioxidant, antimicrobial, and anti-inflammatory capacity [30,31]. Because of these properties, LEO has already been described as a wound-healing enhancer due to its capacity to accelerate wound contraction [3,32,33].

In this work, the preparation of membranes of ALG, CHT, and the combination of both CHT/ALG in which LEO is incorporated, was carried out in order to test their applicability in wound healing. Additionally, its cytotoxicity and cell healing ability were tested in vitro.

## 2. Results and Discussion

The obtained results are presented and discussed below. The first part includes the results related to membrane synthesis and characterization, the study of the corresponding water absorption capacity, and the membranes’ morphology. The second part encompasses the results of the biological characterization of the membranes, which were analyzed in terms of cytotoxicity, which will provide information on cell proliferation and consequent cell regeneration capacity.

### 2.1. Preparation of Membranes

**Figure 1:**
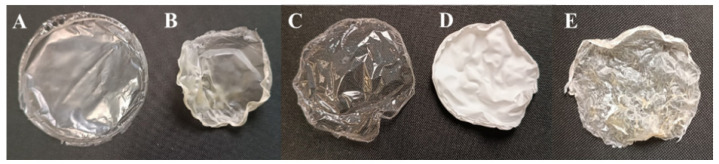
Prepared membranes. (**A**): CHT, (**B**): CHT + LEO, (**C**): ALG, (**D**): ALG + LEO, (**E**): CHT/ALG + LEO.

ALG, ALG + LEO, CHT, and CHT + LEO membranes were obtained. The membranes combining both polymers, CHT/ALG, were not obtained, as they were very fragile, and it was not possible to obtain a complete piece with a homogeneous appearance. However, the CHT/ALG + LEO membranes were successfully prepared and therefore included in all subsequent analyses. Figure 1 shows images of the obtained membranes. The biopolymer membranes that do not contain LEO are more transparent, while when LEO is added, they become opaque. In this regard, it is noteworthy that the ALG membranes become white when LEO is added. The CHT membrane is softer and less wrinkled than the ALG membrane when compared with other non-LEO membranes, which may be related to its lower thickness.

The physical appearances of ALG and CHT membranes are similar to those reported by other authors. S. Ma et al. corroborate the appearance of the CHT films and their transparency [34], and in the work of B. Mutlu et al., it is shown that the incorporation of natural extracts produces a noticeable color change in the films, making them more opaque, as well as that shrinkage and curl occur in the dried film simples [35].

In the case of the combined membrane, CHT/ALG + LEO, its rough character is evident. This may be due to the mixture of both polymers, and it is not necessarily a negative characteristic. At the surface level, it can be said to have an intermediate appearance between the ALG + LEO and CHT + LEO membranes. This aspect is very similar to that proposed by A.P. Rodrigues et al. [22].

### 2.2. Chemical Characterization: Fourier Transform Infrared (FTIR)-Attenuated Total Reflectance (ATR) Spectroscopy

**Figure 2:**
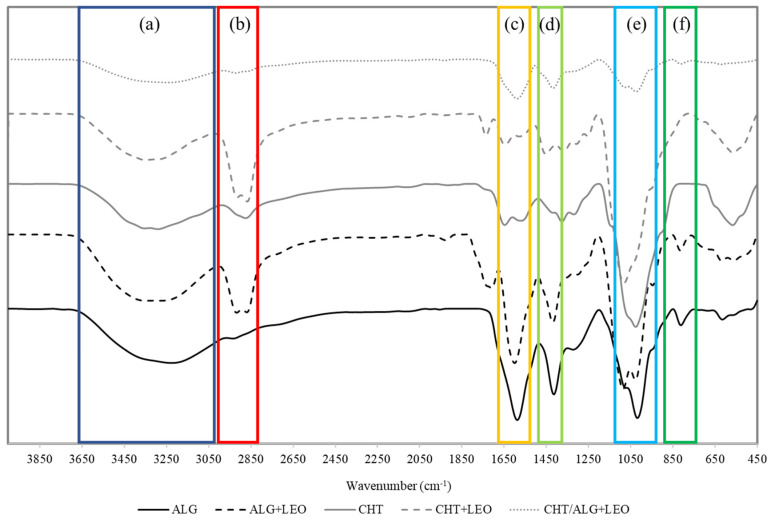
FTIR-ATR spectra of ALG, ALG + LEO, CHT, CHT + LEO, and CHT/ALG + LEO membranes. Wavenumbers marked in dark blue (**a**) correspond to OH stretching region. Wavenumbers marked in red (**b**) correspond to C−H stretching band of CH_2_ groups. Wavenumbers marked in yellow (**c**) correspond to C=C stretching region. Wavenumbers marked in light green (**d**) correspond to asymmetric and symmetric stretching vibrations of carboxylate groups. Wavenumbers marked in light blue (**e**) correspond to the C=O stretching region. Wavenumbers marked in dark green (**f**) correspond to Na-O band.

The spectra corresponding to the FTIR-ATR analysis of the prepared membranes are in Figure 2.

One of the most significant peaks for all samples can be seen around 3000 cm^−1^ due to the strong and broad OH stretching of the hydroxyl group, which overlaps the N-H stretching of chitosan in the same region. Another characteristic peak of sodium alginate is observed at 850 cm^−1^ (Na-O band) [17]. The bands around 1600 cm^−1^ and ~1500 cm^−1^ are associated with asymmetric and symmetric stretching vibrations of carboxylate salt ions [36]. The peak at 1100 cm^−1^ can be assigned to C=O stretching.

Regarding the addition of LEO, the main difference is the broadening of the C-H stretching band of CH_2_ groups to a wavelength of approximately 2900 cm^−1^ (Figure 2). The peak at 1630 cm^−1^ is associated with C=C stretching in components such as linalool, linalyl acetate, terpene-4-ol, and β-caryophyllene abundant in the LEO composition [37]. In the case of CHT, more subtle peaks are observed, but these results are consistent with other published work [38]. From the morphology characterization, it can be concluded that LEO incorporation into the membranes does effectively take place.

### 2.3. Study of Water Uptake and Degradation

**Figure 3:**
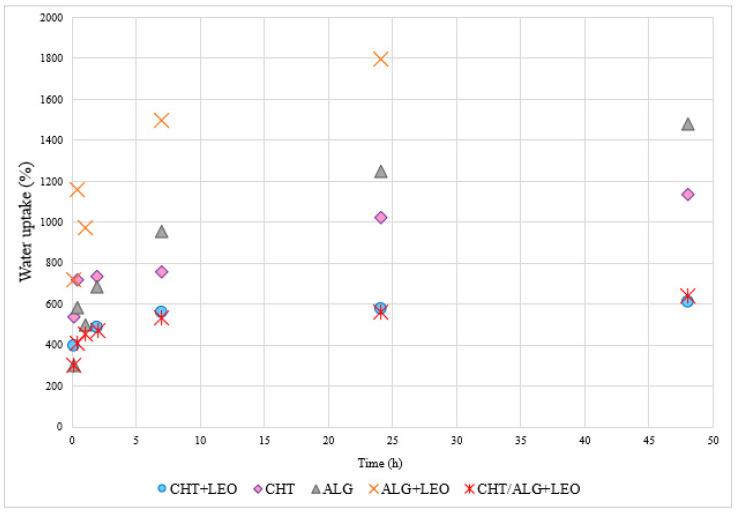
Water uptake ability of membranes over 48 h.

Figure 3 shows the data corresponding to the evaluation of the water uptake capacity of membranes with and without LEO. In the CHT and CHT + LEO membranes, the water uptake increases as time progresses, and the absorption capacity in the first two hours increases rapidly. Comparing the CHT film and CHT + LEO film, it can be observed that the CHT film shows a higher water uptake capacity, compared with the CHT + LEO. This is due to the presence of the LEO, which is hydrophobic and hinders water uptake [31]. The maximum values of the water uptake percentages for the CHT and CHT + LEO membranes take place at 48 h and are 1136% and 606%, respectively. Regarding the integrity of the membranes during this study, it was observed that the CHT and CHT + LEO membranes did not degrade after being immersed in PBS for 48 h. However, after this period they were found to become more fragile as they became softer and wrinkled.

As in the case of the membranes using CHT, the increase in the water uptake percentage during the first two hours in ALG and ALG + LEO films occurs very fast. The water uptake value of ALG continues to increase up to 48 h. It should be noted that the ALG + LEO membranes degraded after 7 h and, as the initial piece of film was found to be divided into small parts, it was not possible to continue with the measurement in this case. After 7 h, the ALG + LEO membrane was able to absorb 1800% of water, while for the ALG film 1477%, it could be quantified after 48 h. As for degradation, the ALG membranes started to decompose after 48 h. Remnants of the membrane were observed in the PBS solution because they started to break down. In this instance, the sample containing LEO has the strongest ability to capture water throughout the entire test, in contrast to the CHT membranes.

Finally, the water uptake ability was quantified for the membrane mixing the two biopolymers, including LEO and CHT/ALG + LEO. Regarding the pattern of the absorption percentage, in this case, the maximum value, or roughly 637%, is attained at 48 h. Furthermore, it is notable that the membrane behavior of CHT/ALG + LEO matches that of CHT more than any other. These findings are consistent with previous research on the water absorption capacity of films made of CHT and ALG [22].

Due to the hydrophilic properties of the two biopolymers utilized, ALG and CHT, all the membranes synthesized had satisfactory water absorption capacities, offering high percentages. The behavior of the contained solutes directly influences this water absorption capacity; the lower the water uptake percentage, the easier it will be for the LEO to be released because it will be less able to become trapped in the membrane [39]. If the films are to be utilized for skin healing and regeneration, this would be advantageous. The strength of the film could also be compromised by excessive water absorption, as is the case with the ALG + LEO film [40]. The CHT/ALG + LEO membranes underwent some degradation and began to behave more like alginate membranes.

### 2.4. LEO Release Profile

**Figure 4:**
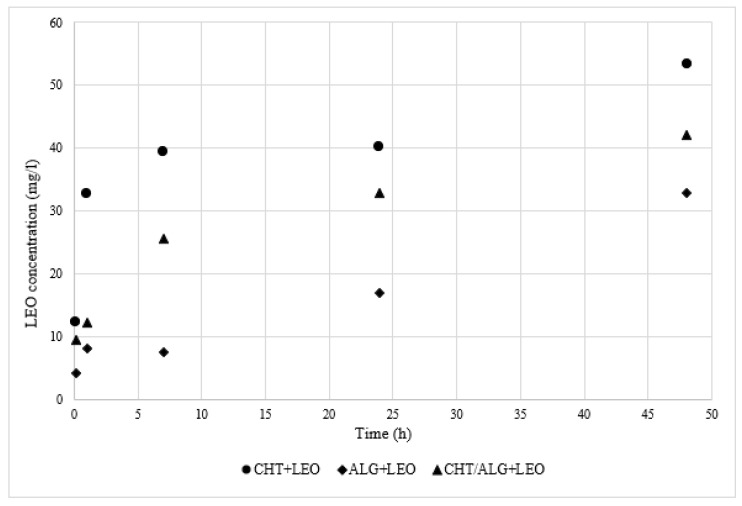
LEO release profile of membranes in PBS at 37 °C for 48 h.

The essential oil release study will help us to understand, on one hand, how fast LEO is released to the physiological-like medium (PBS) and, on the other hand, will help us to better understand cell behavior upon exposure to the membranes on further biological assays. Figure 4 shows the LEO concentration released to PBS for the different membranes.

First, as shown in Figure 4, in all cases, the LEO concentration in PBS increases progressively over the time frame of the assay. Moreover, the rapid increase of this value during the first hour of the tests is remarkable. Oil release slowed down after 1 h due to the reduction of LEO in the membrane section. The release profile is in agreement with the results of similar studies published by other authors [41,42].

On the other hand, comparing the results of the LEO release profile for the different membranes, the CHT film is the one that releases the highest amount of LEO to the PBS, while the ALG film is the one with the lowest LEO concentration values in PBS. These data are in agreement with the results of the water uptake study. The CHT + LEO film had the smallest uptake ability (Figure 3) and offered the highest concentration of oil released (Figure 4), while the opposite was found for the ALG + LEO membrane (Figure 3).

It could be hypothesized that an increased release of LEO may likely lead to a greater reduction in bacterial infection, as LEO has antibacterial potential, and may also likely lead to a decrease in pain associated with injuries, as LEO has been reported to have analgesic activity. To corroborate this, further biological studies need to be performed.

### 2.5. Biological Analysis of the Most Promising Membranes

Since CHT, ALG, and LEO have already been described as wound-healing enhancers through their capacity to accelerate wound contraction and due to their antimicrobial, analgesic, and anti-inflammatory properties [3,17,23], biological analyses were performed to evaluate the wound-healing potential of the membranes.

The CHT/ALG + LEO membrane is considered the most suitable since its water uptake ability and LEO release are intermediate between the CHT + LEO and ALG + LEO membranes and it does not suffer degradation. In addition, the combination of both biopolymers together with LEO is considered a novel proposal that could combine the advantages of both CHT and ALG.

The biocompatibility of the synthesized membranes was studied using a HaCaT cell line as a model to evaluate skin cells’ behavior when in contact with the membranes. Herein, biocompatibility is discussed as a combined result of the effect of membrane leachables on cell viability, cell adhesion to the membranes, and consequent DNA quantification, compared with a control representing the optimal conditions for HaCaT in vitro cell growth.

#### 2.5.1. Cell Viability Assessment

**Figure 5:**
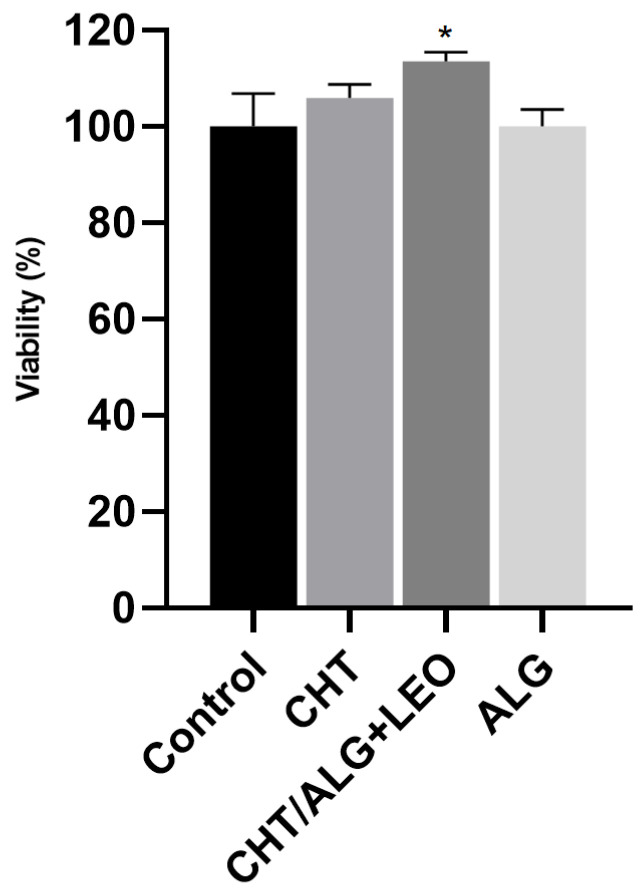
Cells’ viability after exposure to membrane leachables. Results obtained from in vitro indirect contact assay of HaCaT cell line after exposure to the medium that was in contact with the membranes in study over 24 h. The experiment was performed in triplicate, and data are indicated as mean and SD. * *p* < 0.05, as the statistical significance compared with the control.

The results obtained from the leachable assay are represented in Figure 5. The CHT/ALG + LEO membrane was herein studied since it was the one that presented the most interesting characteristics regarding both water uptake and LEO release. Additionally, as controls, also evaluated was the behavior of the CHT and ALG membranes for comparison purposes. In Figure 5, it is possible to observe that the medium collected from the leachables after 24 h of membrane exposure is not toxic to the cells. The average of the results of cell viability obtained for the leachables was 100.1% for the ALG and 106% for the CHT membrane, with the percentage of the CHT/ALG + LEO membrane differing significantly from the control. In fact, the membrane that combines ALG, CHT, and LEO significantly increased cell proliferation by around 10%, suggesting its potential use as a wound-healing enhancer.

#### 2.5.2. Biocompatibility Assays

**Figure 6:**
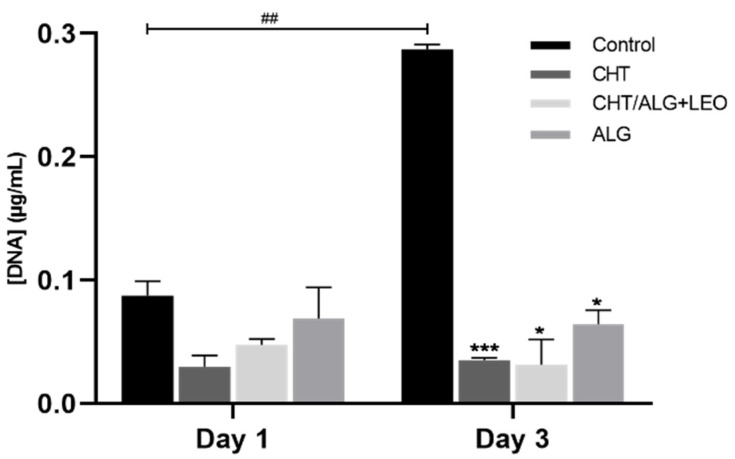
DNA quantification at 1 and 3 days of incubation. Results obtained from the in vitro direct contact assay of HaCaT cells cultured on the surface of the synthesized membranes. The experiment was performed in triplicate and data are indicated as mean and SD. * *p* < 0.05, *** *p* < 0.0005, as the statistical significance compared with the control. ## *p* < 0.005, as the statistical significance compared between controls.

Since a significant increase in cell viability was observed in the indirect cell contact assessment via membrane leachables for the CHT/ALG + LEO, the study moved forward to a direct contact assessment towards HaCaT cells. For that, cells were inoculated directly on top of the different synthesized membrane surfaces, and cell proliferation was measured as a function of the DNA content quantified at two different time points: day 1 and day 3. In Figure 6, it is possible to observe that on day 1, there are no significant differences between the control and any of the tested membranes. Therefore, there were no differences in cell proliferation between the control and cells directly exposed to the different prepared membranes. In contrast, the results obtained upon 3 days of direct exposure revealed not only a significant difference between the control and prepared membranes (but no differences are observed between the membranes on day 1 and on day 3) but also a significant difference between the control of day 3 and control of day 1. These results suggest that there is an impact on HaCaT cells’ proliferation ability upon direct exposure in a time-dependent manner, reflecting that the surface of the membrane is not ideal for cell proliferation.

**Figure 7:**
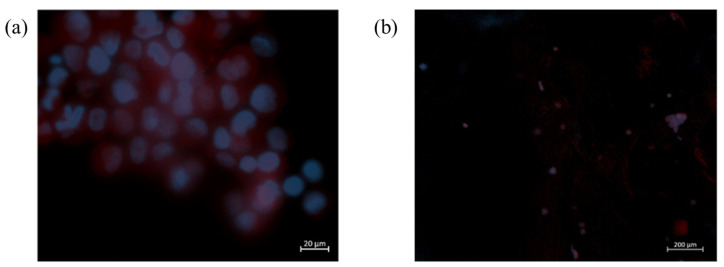
Cell adhesion to the CHT/ALG + LEO membrane after 1 (**a**) and 3 (**b**) days of incubation. Cells were stained with DAPI (blue) to observe the nucleus and phalloidin (red) to observe the actin filaments. Results obtained from the in vitro direct contact assay of HaCaT cells cultured on the surface of the synthesized membranes.

Furthermore, the cell morphology and adhesion to the synthesized membranes were also evaluated, using DAPI and phalloidin fluorescent staining. The obtained results indicate, as expected, that cells seeded on the membranes did not experience cell proliferation (Figure 7).

In summary, the data obtained in the biocompatibility assessment suggest that the indirect contact of the prepared membranes via its leachables does not compromise cell viability and that the cells do not adhere or proliferate on the surface of the membranes. This is an important characteristic because, since the cells do not adhere to the membrane, it may be possible to avoid the breaking of the newly synthesized tissue when the patches are removed from the wounds. Considering the possibility of using such membranes in biomedical applications such as patches for wounds, these results suggest the suitability of CHT/ALG + LEO-based membranes for such applications and demonstrate the possibility of further in vivo testing with living organisms in order to develop products related to this application [43].

## 3. Materials and Methods

### 3.1. Preparation of Membranes

#### 3.1.1. Materials

CHT and Tween 80^®^ were supplied by Sigma-Aldrich (St. Louis, MO, USA). Sodium alginate, ALG, from brown algae was obtained from Fluka-BioChemika (Buchs, Switzerland) and Calcium chloride from PanReac AppliChem (Barcelona, Spain). The LEO was from Peñarrubia del Alto Guadiana S. L. (Albacete, Spain). Acetone, supplied by LabChem (Santo Antão do Tojal, Portugal), acetic acid from Carlo Erba Reagents (Milan, Italy), and deionized water were also used. A total of 20 mM PBS pH 7.4 (including milli Q water, Na_2_HPO_4_.7H_2_O, and NaH_2_PO_4_.H_2_O) was used.

#### 3.1.2. Preparation of Membranes

Membranes were prepared using the method of Rodrigues et al. [22]. For the preparation of the CHT/ALG membranes, 90 mL of a solution of chitosan at 0.5% (*w*/*w*) in 2% aqueous acetic acid (*v*/*v*) and acetone 1:1 (*v*/*v*) was added to 90 mL of an aqueous solution of ALG at 0.5% (*w*/*w*) through a syringe pump (KDS Legato 200 Series) with a flow rate of 40 mL/h and stirring at 500 rpm.

The experiments were carried out at 25 °C in a glass vessel and with a mechanical stirrer. Once the suspension was obtained, it was homogenized for 10 min while stirring at 1000 rpm. Next, the pH was adjusted to 5.28 with the addition of NaOH (1 M) and stirred at 1000 rpm for 10 min. Finally, 1.8 mL of a 2% (*w*/*v*) aqueous CaCl_2_ solution was added for cross-linking. The mixture was then placed in Petri dishes of 15 cm internal diameter and left to dry in an oven with recirculating air for 20 h at 37 °C. After drying, the membranes were immersed in 150 mL of 2% (*w*/*v*) CaCl_2_ aqueous solution for 1 h for cross-linking of alginate L-guluronic acid residues on adjacent chains not bound to chitosan. Then, they were placed in 200 mL of deionized water for 1 h twice and left to dry at room temperature. In the case of membranes composed of a single biopolymer, the same procedure was followed but the first step of mixing the solutions was excluded. The only difference between CHT and ALG membranes is that ALG membranes do not require pH neutralization.

For the preparation of ALG, CHT, and CHT/ALG membranes with essential oil, LEO was added to the ALG solution with a concentration of 1% (*v*/*v*), as well as Tween 80^®^ 1% (*v*/*v*), which acts as an emulsifying agent for the dispersion and solubilization of the essential oil [44].

### 3.2. Determination of Water Uptake and Degradation

In order to test the water uptake capacity of the membranes, a piece of each membrane was immersed in 20 mL of PBS and kept at 37 °C and 60 rpm agitation for 48 h. The membranes were initially weighed on an analytical balance and at specific time intervals after drying with filter paper. Equilibrium water uptake % was calculated from Equation (1) [39,40].

(1) $$\mathrm{Water}\;\mathrm{uptake}\;\%=\frac{W_{w}-W_{d}}{W_{d}}\cdot 100$$

where $W_{w\;}$ is the weight of swollen film and $W_{d}$ is the weight of dried freestanding film.

These tests also assessed the degradation of the membranes in terms of appearance and shape after being immersed for 48 h in PBS. All experiments were carried out in triplicate, and the average result is shown.

### 3.3. Controlled Release of LEO

To evaluate the LEO release process, a piece of each of the ALG + LEO, CHT + LEO, and CHT/ALG + LEO membranes were immersed in 20 mL of PBS. Pieces were cut from each film with weights ranging from 0.012 to 0.06 g. The amount of LEO released was determined by measuring the absorbance at λ = 275nm [42] in a spectrophotometer (Thermo Scientific^TM^ GENESYSTM 50 Vis/UV-Vis Spectrophotometer) of 1 mL sample taken at fixed time intervals (15 min, 1 h, 2 h, 7 h, 24 h, 48 h). The temperature was maintained at 37 °C and after each extraction, the same amount of phosphate buffer saline solution was added to keep the volume constant. Experiments were repeated 3 times and values were calculated as averages.

### 3.4. Morphological Characterization: FTIR-ATR Spectroscopy

FTIR-ATR was used for the identification of functional groups and chemical interactions between the LEO and the film for the different synthesized configurations. A Spectrum Two spectrometer (Perkin Elmer S.L., Madrid, Spain) was used to obtain infrared spectra. The samples were scanned from 4000 to 450 cm^−1^ at a resolution of 16 cm^−1^. All measurements were performed at room temperature.

### 3.5. Cell Culture

With the aim to evaluate the biological performance of ALG, CHT, and ALG/CHT + LEO membranes, HaCaT cell line (German Cancer Research Center (DKFZ), Heidelberg, Germany) was used. HaCaT cells, a human epidermal keratinocyte cell line, were maintained in Dulbecco’s Modification Eagle’s Medium (DMEM, Corning, NY, USA) with phenol red and supplemented with 10% (*v*/*v*) of heat-inactivated fetal bovine serum (FBS, Corning, NY, USA) and 1% (*v*/*v*) of penincilin-strepmycin (PS, Corning, NY, USA). Cell cultures were routinely grown as a monolayer in 75 cm^2^ culture flasks (Falcon, Corning, NY, USA) in a humidified atmosphere at 37 °C with 5% of CO_2_.

### 3.6. Cell Viability Assessment

The cytotoxicity of the membranes was first evaluated by analyzing the effect of their leachables on cell metabolism. For that, cells were seeded in 24-well plates at a density of 1.5 × 10^5^ cells/mL and incubated for 48 h. After the first 24 h, the membranes were cut into small pieces and submerged in culture medium at a concentration of 0.025 g/mL and placed in a 37 °C bath under agitation for 24 h. After, the medium with the leachable was recovered with a syringe and filtered with a 0.45 µm filter. Then, cells were exposed to the leachables in triplicate or to culture medium as a negative control. After 24 h, cell viability was assessed using MTS (3-(4,5-dimethylthiazol-2-yl)-5-(3-carboxymethoxyphenyl)-2-(4-sulfophenyl)-2H-tetrazolium) (16%) (CellTiter 96^®^ AQueous One Solution Cell Proliferation Assay, PROMG3581, Promega, Madison, WI, USA) in a dilution of 1:10 in assay culture medium (DMEM + 0.5% FBS). Cell viability was measured after 3 h by UV-Vis spectroscopy at 490 nm in a microplate reader (HH35L2019044, Victor Nivo 3S, Perkin Elmer, Waltham, MA, USA).

### 3.7. Biocompatibility Assays

After the preliminary cell viability assay, direct contact tests were performed using the same cell line. Prior to cell seeding, membranes were sterilized in UV light for 20 min. After that, membranes were placed on the bottom of 24-well plates in triplicate and cells were seeded (1 × 10^6^ cells/mL) on top of the membranes or in an empty well (control) and incubated for 1 and 3 days at 37 °C with 5% of CO_2_ in a humidified atmosphere. Two different assays were performed, DNA quantification and DAPI-phalloidin staining.

DAPI-phalloidin staining was performed using 4,6-Diaminidino-2-phenylindole-dilactate (DAPI, Corning, NY, USA) and phalloidintetramethylrhodamine B isothiocyanate dyes (phalloidin, Sigma-Aldrich, St. Louis, MO, USA). Briefly, after each time point, cell culture medium was discarded, membranes were washed with PBS (Phosphate buffer saline, Sigma-Aldrich, USA), and cells were fixed with 10% (*v*/*v*) of formalin. After 30 min, formalin was removed and three washes with PBS were performed. After washing, 1 mL of PBS containing 10 µL of phalloidin and 1 µL of DAPI were added for 30 min at room temperature and protected from light. After staining, samples were washed three times with PBS and transferred to a coverslip for fluorescence microscopy observation in an inverted fluorescent microscope (Zeiss, Axio Vert A1, Jena, Germany) with a Colibri 7 (Zeiss, Baden-Württemberg, Germany) light source.

DNA quantification was performed to evaluate cell proliferation on the membranes by quantifying the amount of double-stranded DNA on day 1 and day 3. For that, Quant-IT PicoGreen dsDNA Assay Kit (ThermoFischer, Waltham, MA, USA) was used according to the manufacturer’s instructions. Briefly, culture medium was removed from the wells and replaced by PBS. After two series of PBS washing, membranes were recovered to Eppendorfs and immersed in 1 mL of ultrapure water. After that, the Eppendorfs were placed in a 37 °C bath under agitation for 1h and then stored at −80 °C until use. Samples were then thawed at room temperature and diluted in PicoGreen Solution and 1X TE in a 96-well plate. Samples were placed in triplicate and incubated in the dark for 10 min. Fluorescence was measured in a microplate reader applying an excitation wavelength of 485/20 nm and an emission wavelength of 528/20 nm. The DNA concentration was calculated using a calibration curve.

### 3.8. Statistical Analysis

The statistical analysis was carried out using GraphPad Prism 8.0 (GraphPad Software, San Diego, CA, USA). All biological data are expressed as mean and Standard Deviation (SD), and significant differences were calculated by comparing the different membranes with the control and comparing between membranes. *p*-values smaller than 0.05 were considered statistically significant (confidence interval of 95%). The statistical differences are represented by different numbers of “*” or “#”. To analyze the significant differences, first, the normality of the results was tested using the Shapiro–Wilk test. Since the results did not follow a normal distribution, One-Way ANOVA was used to perform the comparisons.

## 4. Conclusions

The synthesis of biopolymer membranes with ALG, CHT, and CHT/ALG, and the corresponding incorporation of LEO has been successfully developed. FTIR-ATR characterization demonstrated the effectiveness of LEO addition to the different membranes with the characteristic bands of LEO at 2900 cm^−1^ and 1630 cm^−1^. The water uptake capacity study showed that the ALG + LEO film was able to absorb the highest percentage of water, 1797%, but its degradation occurred during the first 7 h, while the rest of the membranes were stable for at least 48 h. In contrast, the CHT + LEO film had the lowest water uptake rate of 600% and released the highest concentration of LEO to the medium during the 48 h of testing. The combined CHT/ALG + LEO film presented water uptake and release capacity results of 638% and 42 mg/L, respectively. These results are positive for CHT/ALG + LEO application in dressings and are supported by the cell viability tests, which showed that the CHT/ALG + LEO membrane leachable increases cell proliferation and are biocompatible, since cells do not grow on the surface of the membrane, avoiding the disruption of the new tissue formed when the membrane is retrieved. These findings open the possibility for more in-depth investigation and antimicrobial testing of these materials. Therefore, CHT/ALG-based membranes are considered suitable for biomedical applications in wound healing.

## Data Availability

The data presented in this study are available on request from the corresponding author. The data are not publicly available due to privacy.
